# Maternal Intake of Vitamin D Supplements during Pregnancy and Pubertal Timing in Children: A Population-Based Follow-Up Study

**DOI:** 10.3390/nu15184039

**Published:** 2023-09-18

**Authors:** Anne Gaml-Sørensen, Nis Brix, Lea Lykke Harrits Lunddorf, Andreas Ernst, Birgit Bjerre Høyer, Gunnar Toft, Tine Brink Henriksen, Cecilia Høst Ramlau-Hansen

**Affiliations:** 1 Department of Public Health, Research Unit for Epidemiology, Aarhus University, Bartholins Alle 2, 8000 Aarhus C, Denmark; 2 Department of Clinical Genetics, Aarhus University Hospital, 8200 Aarhus N, Denmark; 3 Department of Urology, Aarhus University Hospital, 8200 Aarhus N, Denmark; 4 Open Patient Data Explorative Network, Odense University Hospital, 5000 Odense, Denmark; 5 Steno Diabetes Center Aarhus, Aarhus University Hospital, 8200 Aarhus N, Denmark; 6 Department of Clinical Medicine, Aarhus University, 8200 Aarhus N, Denmark; 7 Department of Paediatrics and Adolescent Medicine, Aarhus University Hospital, 8200 Aarhus N, Denmark

**Keywords:** Vitamin D, micronutrient, prenatal exposures, reproductive development

## Abstract

Maternal vitamin D may be important for several organ systems in the offspring, including the reproductive system. In this population-based follow-up study of 12,991 Danish boys and girls born 2000–2003, we investigated if maternal intake of vitamin D supplements during pregnancy was associated with pubertal timing in boys and girls. Information on maternal intake of vitamin D supplements was obtained by self-report in mid-pregnancy. Self-reported information on the current status of various pubertal milestones was obtained every six months throughout puberty. Mean differences in months at attaining each pubertal milestone and an average estimate for the mean difference in attaining all pubertal milestones were estimated according to maternal intake of vitamin D supplements using multivariable interval-censored regression models. Lower maternal intake of vitamin D supplements was associated with later pubertal timing in boys. For the average estimate, boys had 0.5 months (95% CI 0.1; 0.9) later pubertal timing per 5 µg/day lower maternal vitamin D supplement intake. Maternal intake of vitamin D supplements was not associated with pubertal timing in girls. Spline plots and sensitivity analyses supported the findings. Whether the observed association with boys’ pubertal timing translates into an increased risk of disease in adulthood is unknown.

## 1. Introduction

Vitamin D is a prohormone essential to humans [1,2]. Once activated, it exhibits its effects through binding to the vitamin D receptor (VDR) and thereby regulates cell proliferation and differentiation [3]. In addition to well-described effects in bone metabolism, vitamin D has been found to be involved in the function of several other organ systems [1], including the reproductive system [4,5,6].

Vitamin D supplementation during pregnancy may be associated with different pregnancy outcomes, such as hypertensive disorders, gestational diabetes mellitus and risk of transient osteoporosis of the hip [4,7]. Moreover, this may, in turn, be associated with adverse neonatal outcomes, including low birth weight and preterm birth, though results are not conclusive [4].

However, maternal vitamin D supplementation during pregnancy may also directly affect the developing fetus [8]. Maternal and fetal vitamin D levels are highly correlated, and the fetus relies on an adequate maternal supply of vitamin D through the placenta [9]. It has been hypothesized that low maternal vitamin D may have a programming effect on long-term health in children [8,10], potentially by introducing epigenetic alterations [8,10,11]. The hypothalamic–pituitary–gonadal (HPG) axis, which is the key regulator of reproductive maturation [12], may be vulnerable to interferences in fetal life [10,13,14,15], where the reproductive organs develop, and the HPG axis is active [8,16]. The VDR and vitamin D activating enzymes are expressed throughout the HPG axis [5,17], and vitamin D is metabolized in the developing gonads [5,6], suggesting a local role of vitamin D during development. An animal study found that female offspring of vitamin D-depleted mothers had larger ovaries and altered ovarian physiology [18], and in an epidemiologic study, we found that men exposed to low maternal vitamin D levels in utero had lower testes volume compared to men exposed to >75 nmol/L 25(OH)D_3_ levels [19].

Altered pubertal timing is of public health concern due to the associations with morbidity in adulthood, such as cardiovascular, metabolic and psychiatric diseases [20,21]. Therefore, the identification of potential modifiable factors is warranted. No or low maternal intake of vitamin D supplements during pregnancy may represent such a modifiable factor; however, no epidemiologic studies have investigated this. We aimed to investigate the association between maternal intake of vitamin D supplements and pubertal timing in boys and girls from a large population-based puberty cohort.

## 2. Materials and Methods

This population-based cohort study is based on the Danish National Birth Cohort (DNBC) and its sub cohort the Puberty Cohort [22,23].

From 1996 to 2002, pregnant women were invited to the DNBC at their first antenatal visit. Approximately half of all general practitioners in Denmark participated in the enrolment, and around 92,000 women (participation rate: 60%; corresponding to 30% of all pregnancies in Denmark during the recruitment period) were recruited to the DNBC. The study population consisted of Danish-speaking women, as this was an inclusion criterion for invitation. Moreover, the women were primarily Caucasian. At enrolment, around gestational week (GW) 8, the women filled out a form on their current intake of supplements. The women answered questions on health and health behavior in a computer-assisted telephone interview scheduled in GW 12 and completed a validated, semi-quantitative food-frequency questionnaire (FFQ) including a section on the intake of supplements around GW 25 [23]. Follow-up questionnaires were completed when the children were 7 and 11 years old.

**Figure 1:**
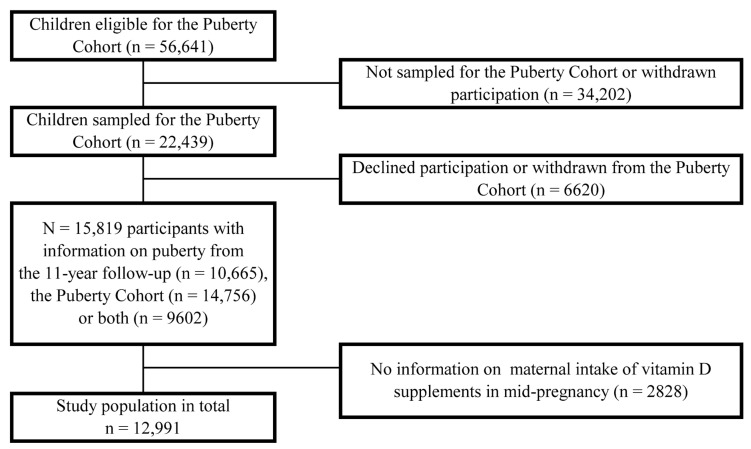
Flow diagram of the inclusion of study participants, the Puberty Cohort, 2000–2021, Denmark.

Live-born singletons born between 2000 and 2003 of mothers that participated in the first DNBC interview, were sampled for the Puberty Cohort. In total, 22,439 children of 56,641 eligible children were sampled as previously described [24]. From the age of 11.5 years, 14,756 of the 22,439 invited children answered web-based questionnaires half-yearly on their current stage of pubertal development. Further, 10,665 of the 22,439 invited children provided information on their current stage of pubertal development during the 11-year follow-up. When combining this data, 15,819 children provided information on pubertal development at least once (participation rate: 70%) [22]. Of those, 12,991 mothers had provided information on the intake of vitamin D supplements in the DNBC FFQ, thereby constituting our final study population (Figure 1).

### 2.1. Vitamin D Supplements

The main exposure was maternal intake of vitamin D supplements including prenatal vitamins in mid-pregnancy, which was obtained from the DNBC FFQ. Each woman was asked about any intake of supplements, including the name, the producer, frequency of intake and the daily dose taken of the potential supplements in the four weeks prior to completion of the questionnaire. Information on the nutrient content of each specific supplement was obtained from the Danish Veterinary and Food Administration, the Danish Medicines Agency, or the producers of the product. Based on this material, the average daily intake of vitamin D supplements in µg was derived from vitamin D dose and frequency of intake during the last four weeks [25]. The FFQ can be found at https://www.dnbc.dk/data-available/food-frequency-questionnaire (accessed on 14 September 2023).

Information on dietary intake of vitamin D was obtained from the FFQ and information on maternal intake of vitamin D supplements in early pregnancy was obtained from the DNBC enrolment form and used in sensitivity analyses.

### 2.2. Pubertal Timing

Information on the current age at attaining numerous pubertal milestones was derived from the Puberty Cohort. The milestones included Tanner Stages 2–5 for genital and pubic hair development [26] in boys and Tanner Stages 2–5 for breast and pubic hair development [27] in girls. The Tanner Stages were obtained by the Sexual Maturation Scale. In boys, information on the age at first ejaculation (in years and months) and voice break (yes, partly, no) was further obtained. In girls, information on the age at menarche (in years and months) was obtained. In both boys and girls, information on axillary hair development (yes, no) and acne (yes, no) was further obtained. Questionnaires are available at https://www.dnbc.dk/data-available/puberty-follow-up (accessed on 14 September 2023).

### 2.3. Covariates

Potential confounding factors were identified a priori using directed acyclic graphs [28] (Supplementary Figure S1) and existing literature and included the following: maternal age at menarche, maternal pre-pregnancy body mass index (BMI), and highest parental socioeconomic status, parental cohabitation, couple fecundity, maternal smoking, maternal alcohol intake, parity, maternal age at delivery and season at delivery (Table 1). The highest parental socioeconomic status was defined according to both level of education and occupation and was derived from the Danish International Standard Class of Occupation and Education codes (ISCO-88 and ISCED). From the Danish Medical Birth Registry (DMBR), data on maternal age at delivery, parity and season at delivery were obtained, while the remaining covariates were obtained from the first DNBC interview.

Information on birth weight and gestational age were also obtained from the DMBR, information on childhood BMI was obtained from the 7-year DNBC follow-up, and information on the mothers’ overall diet quality assessed as a healthy eating index (described in detail in Bjerregaard et al. [29]) was obtained from the mid-pregnancy DNBC FFQ and used in sensitivity analyses.

### 2.4. Statistical Analysis

Mean age differences in months with 95% confidence intervals (CI) at attaining each pubertal milestone according to exposure groups were estimated using a multivariable regression model for censored, time-to-event data (STATA’s intreg package) since the outcome data were censored. In addition, the average mean age at attaining all the pubertal milestones was combined into one model using Huber–White robust variance estimation, to provide an overall estimate for pubertal timing and to account for the risk of type I errors due to multiple testing of correlated outcomes [30,31].

First, to explore potential dose-response associations we estimated the association per 5 µg/day decrease in maternal intake of vitamin D supplements and the pubertal milestones. Second, we estimated the association according to categories of maternal intake of vitamin D supplements (0 µg; 0.01–9.99 µg; ≥10 µg) with ≥10 µg/day (range: 10–41 µg/day) as the reference group. Third, we modeled restricted cubic spline plots with three knots (at the 10th (0 µg/day), the 50th (7.14 µg/day), and the 90th (10 µg/day) percentiles) to visualize the associations between maternal intake of vitamin D supplements and the overall estimate for pubertal timing.

We performed six sensitivity analyses using the overall estimate for pubertal timing as the outcome.

First, we considered the total intake of vitamin D (µg/day) by adding the estimated vitamin D intake from the diet as assessed in the FFQ to the intake from vitamin D supplements also obtained from the FFQ. Information on dietary intake of vitamin D was obtained from the validated, semi-quantitative FFQ, which consisted of 360 items of food and beverages. Daily nutrient intake in µg was derived based on estimated standard recipes, standard portion sizes and the Danish food composition tables including nutrient content [32] as described in detail previously [25]. We estimated the potential linear association per 5 µg/day decrease in total intake of vitamin D and total intake of vitamin D categorized in quartiles. Dietary intake of vitamin D was not integrated into the main analysis, as this may correlate poorly with actual intake due to measurement errors when using an FFQ [33], and since dietary intake of vitamin D may play a minor role in bioavailable plasma vitamin D [1].

Second, we adjusted for a healthy eating index, which was a composite score of diet quality based on the current Danish Food-Based Dietary Guidelines, as described previously [29]. In short, a high score indicated high compliance with the current guidelines and, hence, a high-quality diet. The score was based on a high intake of fruits and vegetables, dietary fibers, and fish, and low intakes of red meat, saturated fatty acids, sodium, sugar-sweetened beverages and added sugar. Since the healthy eating index was not available for all participants, we further restricted the main analysis to comprise those with information on the healthy eating index (*n* = 7562) but without further adjusting for the score, to explore potential selection bias due to missing information.

Third, we did a simple mediation analysis by further adjusting the main models for birth weight z-scores [9] that is the number of standard deviations (SDs) that birth weight differed from the expected birth weight given sex and gestational age using the growth curves from Marsál et al. [34].

Fourth, we considered potential mediation by childhood BMI (continuous in kg/m^2^) [35] at age 7 years, by including this in the main model. The risk of bias due to missing information because of attrition in the 7-year follow-up wave in the DNBC was assessed by also restricting the main analysis to comprise only participants having information on childhood BMI (*n* = 9573).

Fifth, we divided the reference group of women having an intake of ≥10 µg/day into two groups (women having an intake of >10 µg/day in one and women having an intake of 10 µg/day in another) to explore potential confounding by indication in the group with the highest intake of vitamin D supplements.

Sixth, we explored early pregnancy exposure to maternal intake of vitamin D supplements compared to maternal intake of no supplement or other supplements. Information on maternal intake of vitamin D supplements in early pregnancy (around gestational week 8) was obtained from the enrolment form in the DNBC. Each woman was asked whether she took any supplements, including the name of the product. Based on the nutrient content of this, a categorized variable was derived (no supplements, other supplements not containing vitamin D, multivitamin supplements also containing vitamin D, and vitamin D supplements with or without calcium). Women using several types of supplements were categorized according to the supplement containing the highest amount of vitamin D. Information on the nutrient content of each specific supplement was obtained from the Danish Medicines Agency, the Danish Veterinary and Food Administration or the producers of the product.

All models were adjusted for the selected covariates. To consider the sampling strategy used in the Puberty Cohort, we fitted all models with inverse probability of sampling weights, as described previously [24]. To consider a risk of potential selection bias due to selective non-participation, we calculated and fitted all models with inverse probability of selection weights. These selection weights corresponded to the inverse probability of participation [36]. For the main and each sensitivity analysis, selection weights were calculated based on the exposure of interest in addition to the identified covariates as explanatory variables for participation. All models were further fitted with robust standard errors to account for the use of the weights and for the clustering of siblings.

All analyses were conducted assuming normally distributed residuals. In R (x64 3.3.1), we compared the non-parametric cumulative incidence function, based on the Turnbull Estimator, with the normal distribution [37,38]. The data were compatible with the assumption. All percentiles were calculated as the mean of the five values nearest to the actual percentile (pseudo percentiles) due to local regulations (GDPR, Regulation (EU), 2016/679 of 25 May 2018). Data management and statistical analyses were conducted in STATA 17.0 (Statacorp, College Station, TX, USA).

## 3. Results

In total, 1977 (15%) women had no intake of vitamin D supplements in mid-pregnancy, 4823 (37%) women had an intake of vitamin D supplements of 0.01–9.99 µg/day, and 6191 (48%) women had an intake of ≥10 µg/day. Median intake of vitamin D supplements in mid-pregnancy was 7.14 µg/day (range 0–41). Women without any intake of vitamin D supplements in mid-pregnancy (15%) were more likely to be multipara, to have an unplanned pregnancy, and to smoke during the first trimester compared to women reporting an intake of vitamin D supplements, and women with an intake of ≥10 µg/day (48%) were more likely to be primipara, to have >12 months TTP or use of MAR, and were less likely to drink alcohol (Table 1).

**Figure 2:**
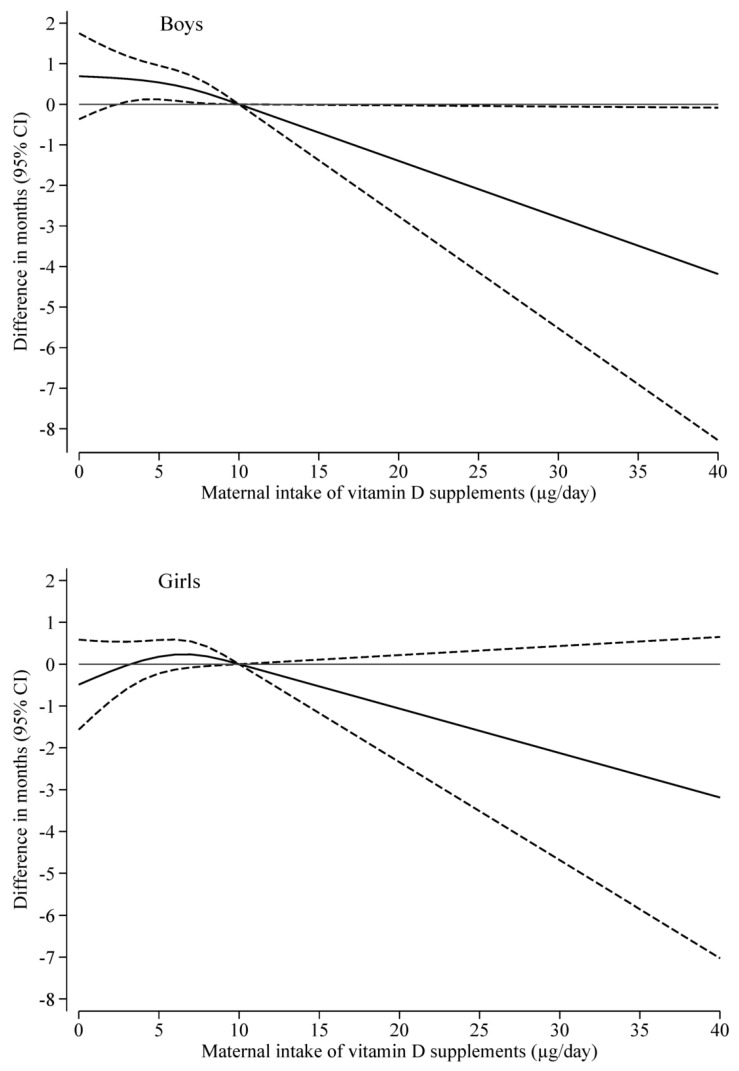
Restricted cubic spline plots with three knots for the overall estimate for pubertal timing according to maternal intake of vitamin D supplements in mid-pregnancy (solid lines) with 95% confidence intervals (dotted lines), relative to a maternal intake of vitamin D supplements of 10 µg/day. Plots are adjusted for maternal age at menarche, maternal pre-pregnancy body mass index, and highest parental socioeconomic status, parental cohabitation, couple fecundity, maternal smoking, maternal alcohol intake, parity, maternal age at delivery and season at delivery. Top panel shows associations for boys (*n* = 6017) and bottom panel shows associations for girls (*n* = 6430).

Lower maternal intake of vitamin D supplements in mid-pregnancy was associated with later pubertal timing in boys in a dose-dependent manner (Table 2) for all individual pubertal milestones, although some confidence intervals overlapped the null. For the overall estimate for pubertal timing, boys had 0.5 months (95% CI: 0.1; 0.9) later pubertal timing per 5 µg lower maternal intake of vitamin D supplements per day. The categorical analysis and spline plot (*p* = 0.04) supported these findings (Table 2 and Figure 2).

Maternal intake of vitamin D supplements in mid-pregnancy was not associated with pubertal timing in girls (overall estimate for pubertal timing was 0.1 months (95% CI: −0.3; 0.5) later pubertal timing per 5 µg lower maternal intake of vitamin D supplements per day) (Table 2 and Figure 2).

Results remained essentially the same in all sensitivity analyses investigating mid-pregnancy maternal intake of vitamin D supplements. When considering total intake of vitamin D both from supplements and food, lower maternal vitamin D intake was associated with later pubertal timing in boys (combined estimate 0.5 months (95% CI: 0.1; 0.8 months) per 5 µg lower total intake of vitamin D per day), but not in girls (combined estimate 0.1 months (95% CI: 0.3; −0.4 months) per 5 µg lower total intake of vitamin D per day). Results remained essentially similar in the subanalyses further adjusting for the healthy eating index, birth weight z-scores, and when investigating the women with an intake of >10 µg/day as a separate exposure group. When further adjusting for childhood BMI, results attenuated slightly in boys (combined estimate 0.3 months (95% CI: −0.1; 0.7 months) per 5 µg lower total intake of vitamin D per day). In the sixth subanalysis investigating maternal intake of supplements in early pregnancy, no maternal intake of supplements was suggestive of later pubertal timing in boys (overall estimate: 1.2 months (95% CI: −0.2; 2.5 months)) and suggestive of earlier pubertal timing in girls (overall estimate: −0.9 months (95% CI: −2.2; 0.4 months)) compared to boys and girls of mothers reporting intake of vitamin D supplements (Table 3).

## 4. Discussion

We found that lower maternal intake of vitamin D supplements in mid-pregnancy was associated with slightly later pubertal timing in boys. We found no consistent association between mid-pregnancy maternal intake of vitamin D supplements and pubertal timing in girls. The results were robust across sensitivity analyses.

### 4.1. Strengths and Limitations

The major strength of this study is the longitudinal design with detailed information on maternal intake of vitamin D supplements during pregnancy, various pubertal milestones throughout pubertal development and many potentially important confounding and mediating factors. The large sample size limited the risk of type II errors, and we were able to adjust for many important potential confounders measured at baseline; e.g., maternal pre-pregnancy BMI, which is an important factor in determining vitamin D bioavailability, since vitamin D is stored in adipose tissue [39]. Due to the observational design applied in this study, we cannot, however, eliminate the risk of residual confounding.

The participation rate was high (70%). Participation in the Puberty Cohort was not associated with a marker of pubertal timing (the height difference in standard deviations (HD:SDS)), obtained from an external registry [40], and maternal intake of vitamin D did not differ between participants and non-participants (Supplementary Table S1), indicating limited risk of selection bias.

Children were followed-up half-yearly throughout puberty, limiting the risk of potential recall problems. In addition, the validity of the self-assessment in the puberty cohort has been found to be fair to moderate [41]. Any potential misclassifications are likely to be non-differential regarding maternal vitamin D supplement intake.

Information on maternal intake of vitamin D supplements was self-reported and may suffer from non-differential misclassification, inducing potential bias towards the null. Further, reported intake of vitamin D supplements may not reflect vitamin D bioavailability, since this is dependent on the dissolvability of the supplement and administration of the supplement alongside other foods [42]. However, maternal intake of vitamin D supplements during pregnancy does increase bioavailable vitamin D (25-hydroxyvitamin D (25(OH)D)) at term [43]. Moreover, we found a positive, albeit not strong, correlation between maternal intake of vitamin D supplements in mid-pregnancy and plasma levels of vitamin D (25(OH)D_3_) in another subset of the DNBC (Supplementary Text S1) [44].

Whether the average intake of vitamin D supplements in mid-pregnancy applies to the entire pregnancy was unknown. However, mid-pregnancy vitamin D supplement intake differed significantly according to early pregnancy intake of supplements (Supplementary Table S2), indicating that the pregnant women may have had a stable pattern of supplement intake throughout the pregnancy. E.g. women reporting no intake of supplements in early pregnancy had a significantly lower intake of vitamin D supplements assessed in mid-pregnancy compared to women reporting intake with vitamin D supplements with or without calcium in early pregnancy.

### 4.2. Interpretation

No published epidemiologic study has investigated the association between intake of vitamin D supplements in pregnancy and offspring pubertal timing. In a study in mice, investigating female offspring only, maternal vitamin D depletion did not affect pubertal timing [18], which is in line with our results. However, maternal vitamin D depletion was associated with other markers of impaired reproductive health, such as altered ovarian physiology, characterized by lower luteinizing hormone secretion and oligo ovulation, potentially induced by hypothalamic dysfunction [18].

In another study from the Puberty Cohort, we have previously found an association between maternal 25(OH)D_3_ levels predicted based on the season of the first pregnancy trimester and earlier pubertal timing in both boys and girls [45]. In the study using season of gestational week 8 as an instrumental variable for maternal 25(OH)D_3_ levels, we found that boys experienced earlier pubertal timing of −1.0 months (95% CI: −1.8; −0.2 months) per 22 nmol/L lower maternal 25(OH)D_3_ levels for the combined estimate for pubertal timing. Girls experienced earlier pubertal timing of −1.3 months (95% CI: −2.1; −0.4 months) per 22 nmol/L lower maternal 25(OH)D_3_ levels for the combined estimate for pubertal timing [45]. This inconsistency warrants further exploration, but could be explained by potential violation of the assumptions underlying the applied methods or may arise due to bias. However, the inconsistency may also be explained by the two different exposures under investigation (maternal intake of vitamin D supplements in mid-pregnancy vs. maternal 25(OH)D_3_ levels predicted based on season of gestational week 8), or due to investigation of different exposure windows.

We investigated early pregnancy supplement intake in a subanalysis. The exact exposure window was unknown; however, early pregnancy exposure may also be important, since, in addition to cell proliferation, vitamin D also regulates differentiation, which is more prominent in the early phase of gestation [3]. The subanalysis of vitamin D supplements in early pregnancy was suggestive of slightly earlier pubertal timing in girls and later pubertal timing in boys of mothers reporting no use of supplements in early pregnancy compared to mothers, who reported intake of vitamin D supplement. Importantly, compared to boys and girls of mothers, who reported use of other supplements or multivitamins, we observed no differences in pubertal timing, suggesting that the difference may be attributable to taking prenatal vitamins or not, which could be a result of residual confounding.

Worldwide, there are no consistent recommendations regarding the intake of vitamin D supplements during pregnancy [4,9]. The World Health Organization recommends pregnant women engage in sensible sun exposure and a balanced diet [46], while the Institute of Medicine and the US Endocrine Society recommend a total intake of 15 µg/day and 25–50 µg/day respectively from dietary sources including vitamin D supplements [9]. In our study, where the women were pregnant before the current recommendations were deployed, the mean total intake of vitamin D from dietary sources was 10.7 (SD: 5.2). Even after the official recommendation of 10 µg vitamin D per day throughout pregnancy was implemented in 2009 in Denmark [47], many Danish pregnant women may still suffer from low vitamin D levels [48]. This trend of low vitamin D levels in pregnant women is observed worldwide, why low vitamin D during pregnancy is a public health concern, not only for the reproductive health of the offspring [1,4,5].

Our study population consisted of women and children of Western origin in a country with seasonal variations in skin synthetization of vitamin D and without any food fortification with vitamin D. Whether the impact of maternal intake of vitamin D supplements may be different in populations of other ancestries, in populations living at lower latitudes, or in populations where foods are fortified with vitamin D remain to be settled. Overall, our results may be generalizable to similar populations.

## 5. Conclusions

We observed slightly later pubertal timing in boys of mothers with no or low intake of vitamin D supplements in mid-pregnancy compared to boys of mothers having a higher intake of vitamin D supplements. Whether this minor delay is clinically relevant from a public health perspective is unknown. Maternal intake of vitamin D supplements was not associated with pubertal timing in girls.

## Figures and Tables

**Table 1:** Distribution of covariates according to maternal intake of vitamin D supplements (µg/day) in mid-pregnancy among 12,991 children from the Puberty Cohort Denmark 2000–2021.

		Average Daily Intake of Vitamin D from Supplements (µg/day)			
		≥10	0.01–9.99	0	Missings
		*n* = 6191 (48%)	*n* = 4823 (37%)	*n* = 1977 (15%)	
Alcohol 1st trimester (drinks/week) ^b^					>20 ^a^ (0)
	0	3312 (54)	2326 (48)	<976 ^a^ (49)	
	0.1–1.0	1894 (31)	1617 (34)	612 (31)	
	1.1–3.0	712 (12)	602 (12)	264 (13)	
	> 3.0	263 (4)	268 (6)	125 (6)	
Maternal age at delivery (years (SD))		30.5 (4.3)	31.0 (4.2)	30.5 (4.7)	5 (0)
Couple fecundity ^c^					32 (0)
	Unplanned pregnancy	843 (14)	712 (15)	395 (20)	
	0–5 months TTP	3338 (54)	2670 (55)	1032 (52)	
	6–12 months TTP	806 (13)	621 (13)	248 (13)	
	>12 months TTP + MAR	1193 (19)	806 (17)	295 (15)	
Maternal age at menarche					90 (1)
	Earlier than peers	1538 (25)	1265 (26)	470 (24)	
	Same as peers	3554 (57)	2724 (56)	1135 (57)	
	Later than peers	1059 (17)	796 (17)	360 (18)	
Maternal BMI (kg/m^2^)					175 (1)
	<18.5	399 (6)	326 (7)	131 (7)	
	18.5–≤24.9	3812 (62)	2991 (62)	1176 (59)	
	25–≤29.9	1259 (20)	1009 (21)	410 (21)	
	≥30	638 (10)	434 (9)	231 (12)	
Parental cohabitation					5 (0)
	Yes	<6077 ^a^ (98)	<4740 ^a^ (98)	1930 (98)	
	No	114 (2)	83 (2)	47 (2)	
Parity					0 (0)
	Primipara	3575 (58)	2305 (48)	806 (41)	
	Multipara	2616 (42)	2518 (52)	1171 (59)	
Highest parental socioeconomic status					25 (0)
	High-grade professional	1426 (23)	1280 (27)	416 (21)	
	Low-grade professional	2040 (33)	1662 (34)	595 (30)	
	Skilled worker	1753 (28)	1201 (25)	578 (29)	
	Unskilled worker	816 (13)	559 (12)	319 (16)	
	Student	117 (2)	89 (2)	46 (2)	
	Economically inactive	29 (0)	22 (0)	18 (1)	
Smoking 1st trimester (cigarettes/day)					47 (0)
	0	4514 (73)	3690 (77)	1357 (69)	
	1–10	1355 (22)	897 (19)	457 (23)	
	>10	299 (5)	217 (5)	158 (8)	
Season at birth					0
	Winter	1597 (26)	1190 (25)	528 (27)	
	Spring	1605 (26)	1356 (28)	495 (25)	
	Summer	1546 (25)	1217 (25)	489 (25)	
	Fall	1443 (23)	1060 (22)	465 (24)	
Healthy eating index ^d^ (score (SD))		23.0 (7)	23.0 (7)	22.7 (7)	5429 (42)
Birth weight (grams (SD))		3524 (604)	3556 (588)	3514 (585)	41 (0)
Gestational age at delivery (days (SD))		279 (13)	279 (13)	279 (13)	42 (0)
Child BMI (kg/m^2^)		15.6 (1.7)	15.6 (1.7)	15.8 (1.8)	3418 (26)

Presented as proportions (%) or means (SD). Due to rounding of percentages numbers may not add up to 100%; Abbreviations: BMI, body mass index; SD, standard deviation; TTP, time to pregnancy; MAR, medically assisted reproduction; ^a^ Due to local data regulations it is not allowed to report smaller numbers than five why the numbers in the table have been rounded up or down to mask the numbers smaller than five; ^b^ 1 drink = 12 g of pure alcohol; ^c^ Including unplanned pregnancy, time to pregnancy and medically assisted reproduction; ^d^ Healthy eating index. See text for details.

**Table 2:** Crude and adjusted ^a^ age difference (β) in months (95% CIs) in timing of puberty according to average daily intake of vitamin D supplements. Between 0.01–9.99 µg/day and 0 µg/day relatively to ≥10 µg/day and continuous per 5 µg decrease in vitamin D intake.

		Average Daily Intake of Vitamin D from Supplements (µg/day)						
		≥10 (Reference)	0.01–9.99		0		Pr. 5 µg/day Decrease	
	Pubertal Milestones	Mean Age in Years	Crude Difference	Adjusted Difference	Crude Difference	Adjusted Difference	Crude Difference	Adjusted Difference
	
Boys *n* = 6031 ^b^								
	Tanner Genital stage 2	10.8	0.5	0.9 (−0.3; 2.1)	0.7	1.4 (−0.3; 3.0)	0.5	0.7 (0.1; 1.2)
	Tanner Genital stage 3	12.5	0.2	0.1 (−1.1; 1.2)	0.1	0.5 (−1.1; 2.1)	0.1	0.2 (−0.3; 0.8)
	Tanner Genital stage 4	13.7	0.2	0.0 (−1.2; 1.2)	0.5	1.1 (−0.4; 2.7)	0.2	0.4 (−0.2; 1.0)
	Tanner Genital stage 5	15.8	0.1	0.1 (−1.9; 2.0)	0.0	0.3 (−2.2; 2.8)	0.3	0.7 (−0.3; 1.6)
	Tanner Pubic Hair stage 2	11.3	0.5	0.7 (−0.5; 1.8)	0.7	1.0 (−0.5; 2.6)	0.4	0.5 (0.0; 1.1)
	Tanner Pubic Hair stage 3	12.7	0.4	0.5 (−0.6; 1.5)	0.1	0.6 (−0.7; 1.9)	0.1	0.3 (−0.2; 0.8)
	Tanner Pubic Hair stage 4	13.5	0.6	0.6 (−0.3; 1.6)	−0.1	0.7 (−0.6; 1.9)	0.1	0.4 (−0.1; 0.9)
	Tanner Pubic Hair stage 5	14.8	0.2	0.1 (−1.2; 1.4)	0.1	0.9 (−0.9; 2.6)	0.2	0.5 (−0.1; 1.1)
	Axillary hair	13.2	1.4	1.6 (0.4; 2.9)	−0.1	−0.2 (−1.9; 1.4)	0.2	0.5 (0.0; 1.1)
	Acne	12.2	1.2	1.2 (0.1; 2.4)	1.3	2.1 (0.5; 3.7)	0.7	0.9 (0.4; 1.5)
	Voice break	13.0	0.8	0.6 (−0.6; 1.7)	−0.5	−0.5 (−2.2; 1.1)	0.2	0.2 (−0.4; 0.7)
	First ejaculation	13.3	0.8	1.4 (0.3; 2.6)	0.8	1.2 (−0.4; 2.7)	0.5	0.8 (0.3; 1.4)
	Combined estimate		0.6	0.6 (−0.2; 1.5)	0.3	0.7 (−0.4; 1.8)	0.4	0.5 (0.1; 0.9)
Girls *n* = 6445 ^b^								
	Tanner Breast stage 2	9.8	−0.3	0.4 (−1.2; 2.0)	−0.7	−0.5 (−2.6; 1.6)	−0.4	0.2 (−0.5; 0.9)
	Tanner Breast stage 3	11.6	0.1	0.3 (−0.7; 1.3)	−0.7	−1.0 (−2.3; 0.4)	−0.2	0.0 (−0.5; 0.4)
	Tanner Breast stage 4	13.1	−0.1	0.3 (−0.8; 1.3)	−0.7	−0.5 (−2.0; 0.9)	−0.2	0.0 (−0.5; 0.5)
	Tanner Breast stage 5	16.0	−0.5	−0.2 (−2.2; 1.8)	−3.3	−2.2 (−4.9; 0.5)	−1.0	−0.3 (−1.3; 0.8)
	Tanner Pubic Hair stage 2	11.2	0.2	0.5 (−0.3; 1.4)	0.4	0.4 (−0.7; 1.5)	0.2	0.4 (0.0; 0.9)
	Tanner Pubic Hair stage 3	12.4	0.3	0.5 (−0.3; 1.4)	0.1	0.3 (−0.7; 1.5)	0.1	0.3 (−0.1; 0.7)
	Tanner Pubic Hair stage 4	13.5	0.0	0.4 (−0.7; 1.6)	0.6	0.6 (−0.9; 2.2)	0.3	0.4 (−0.1; 0.9)
	Tanner Pubic Hair stage 5	15.6	−0.3	0.4 (−1.3; 2.1)	0.0	−0.5 (−2.9; 1.9)	−0.1	−0.1 (−1.0; 0.7)
	Axillary hair	11.9	−0.3	0.1 (−1.1; 1.2)	−0.2	0.2 (−1.3; 1.8)	−0.1	0.3 (−0.3; 0.8)
	Acne	11.4	−0.1	−0.1 (−1.5; 1.2)	−1.5	−2.2 (−3.9; −0.4)	−0.5	−0.7 (−1.3; −0.1)
	Menarche	13.0	0.8	0.8 (−0.1; 1.7)	−0.8	−0.7 (−1.9; 0.4)	−0.2	0.1 (−0.4; 0.5)
	Combined estimate		0.1	0.4 (−0.4; 1.2)	−0.5	−0.5 (−1.6; 0.6)	−0.1	0.1 (−0.3; 0.5)

^a^ Adjusted for maternal age at menarche, maternal pre-pregnancy body mass index, and highest parental socioeconomic status, parental cohabitation, couple fecundity, maternal smoking, maternal alcohol intake, parity, maternal age at delivery and season at delivery; ^b^ *n* refers to the number of boys and girls that gave information on all of the pubertal milestones.

**Table 3:** Results from the sensitivity analyses. Crude and adjusted ^a^ age difference (β) in months (95% CIs) for overall pubertal timing.

		Boys			Girls		
		Crude Difference	Adjusted Difference		Crude Difference	Adjusted Difference	
(1) Total intake of vitamin D							
	Highest quartile (13.48−45.65)	Ref.	Ref.		Ref.	Ref.	
	Third quartile (11−38−13.48)	−0.2	0.1 (−1.0; 1.1)		−0.3	0.6 (−0.5; 1.6)	
	Second quartile (7.17−11−38)	0.1	0.5 (−0.5; 1.6)		0.1	1.0 (0.0; 2.0)	
	Lowest quartile (0−7.17)	0.4	0.8 (−0.2; 1.9)		−0.5	0.2 (−0.9; 1.2)	
	Pr. 5 µg/day decrease in vitamin D	0.3	0.5 (0.1; 0.8)		−0.2	0.1 (−0.3; 0.4)	
(2a) Main model further adjusted for healthy eating index							
	≥10 µg	Ref.	Ref.		Ref.	Ref.	
	0.01–9.99 µg	0.7	0.6 (−0.5; 1.7)		−0.3	0.1 (−1.0; 1.1)	
	0 µg	0.5	0.7 (−0.8; 2.2)		−1.0	−0.9 (−2.3; 0.4)	
	Pr. 5 µg/day decrease in vitamin D	0.3	0.4 (−0.1; 0.9)		−0.4	−0.2 (−0.7; 0.3)	
(2b) Main model with restriction to participants with information on healthy eating index							
	≥10 µg	Ref.	Ref.		Ref.	Ref.	
	0.01–9.99 µg	0.7	0.6 (−0.5; 1.7)		−0.3	0.1 (−1.0; 1.1)	
	0 µg	0.5	0.7 (−0.8; 2.2)		−1.0	−0.9 (−2.3; 0.4)	
	Pr. 5 µg/day decrease in vitamin D	0.3	0.4 (−0.1; 0.9)		−0.4	−0.2 (−0.7; 0.3)	
(3) Main model further adjusted for birth weight z−scores							
	≥10 µg	Ref.	Ref.		Ref.	Ref.	
	0.01–9.99 µg	0.6	0.6 (−0.2; 1.4)		0.1	0.3 (−0.5; 1.2)	
	0 µg	0.3	0.7 (−0.4; 1.8)		−0.5	−0.4 (−1.5; 0.7)	
	Pr. 5 µg/day decrease in vitamin D	0.4	0.5 (0.1;0.9)		−0.1	0.1 (−0.3;0.5)	
(4a) Main model further adjusted for childhood BMI at age 7 years							
	≥10 µg	Ref.	Ref.		Ref.	Ref.	
	0.01–9.99 µg	0.6	0.4 (−0.5; 1.4)		−0.1	0.2 (−0.7; 1.1)	
	0 µg	0.3	0.5 (−0.7; 1.7)		−0.6	−0.6 (−1.8; 0.7)	
	Pr. 5 µg/day decrease in vitamin D	0.3	0.3 (−0.1;0.7)		−0.2	0.0 (−0.5; 0.4)	
(4b) Main model with restriction to participants with information on childhood BMI							
	≥10 µg	Ref.	Ref.		Ref.	Ref.	
	0.01–9.99 µg	0.6	0.6 (−0.3; 1.5)		−0.1	0.2 (−0.7; 1.1)	
	0 µg	0.3	0.5 (−0.8; 1.7)		−0.6	−0.7 (−2.0; 0.5)	
	Pr. 5 µg/day decrease in vitamin D	0.3	0.3 (−0.1;0.7)		−0.2	−0.1 (−0.5; 0.4)	
(5) Main model with 10 µg and >10 µg as separate exposure categories							
	>10 µg	−0.3	−0.7 (−2.1; 0.7)		0.6	−0.3 (−1.8; 1.1)	
	10 µg	Ref.	Ref.		Ref.	Ref.	
	0.01–9.99 µg	0.6	0.5 (−0.3; 1.4)		0.2	0.4 (−0.5; 1.2)	
	0 µg	0.3	0.6 (−0.5; 1.7)		−0.5	−0.5 (−1.6; 0.6)	
(6) Early pregnancy supplement intake							
	Vitamin D with/without calcium	Ref.	Ref.		Ref.	Ref.	
	Multivitamin	0.0	0.2 (−1.0; 1.3)		−0.1	0.3 (−0.9; 1.4)	
	Other vitamin	0.3	0.1 (−1.5; 1.7)		0.0	0.1 (−1.5; 1.7)	
	No vitamin	0.5	1.3 (−0.1; 2.7)		−1.5	−1.0 (−2.3; 0.3)	

(1) Pubertal timing according to mid-pregnancy intake of total vitamin D from supplements and diet in µg/day in quartiles with the highest quartile as the reference and per 5 µg/day decrease (*n* = 6017 and for boys *n* = 6430 for girls). (2) Pubertal timing according to mid-pregnancy intake of vitamin D supplements. 2a: Main model further adjusted for healthy eating index. 2b: Main model restricted to participants with information on healthy eating index, without adjusting for healthy eating index (*n* = 3401 boys and *n* = 3917 for girls). (3) Pubertal timing according to mid-pregnancy intake of vitamin D supplements. Main model further adjusted for birth weight z-scores (*n* = 5983 for boys and *n* = 6426 for girls). (4) Pubertal timing according to mid-pregnancy intake of vitamin D supplements. 4a: Main model further adjusted for childhood BMI. 4b: Main model restricted to participants with information on childhood BMI, without adjusting for childhood BMI (*n* = 4541 for boys and *n* = 4672 for girls). (5) Pubertal timing according to mid-pregnancy intake of vitamin D supplements. >10 µg/day, between 0.01-9.99 µg/day and 0 µg/day relative to 10 µg/day (*n* = 6017 for boys and *n* = 6430 for girls). (6) Pubertal timing according to early pregnancy intake of vitamins. No vitamin intake, other vitamin intake, multivitamin intake relative to intake of vitamins with vitamin D with or without calcium (*n* = 7254 for boys and *n* = 7798 for girls). Abbreviations: BMI, body mass index. ^a^ Adjusted for maternal age at menarche, maternal pre-pregnancy body mass index, and highest parental socioeconomic status, parental cohabitation, couple fecundity, maternal smoking, maternal alcohol intake, parity, maternal age at delivery and season at delivery.

## Data Availability

The dataset analysed in the study is not publicly available due to national data security legislation on sensitive personal data. Researchers may, however, apply for access to data from the DNBC. Please see https://www.dnbc.dk/data-available (accessed on 14 September 2023) for additional information.

## Supplementary Materials

The following supporting information can be downloaded at: https://www.mdpi.com/article/10.3390/nu15184039/s1, Supplementary Figure S1: Directed acyclic graph illustrating the assumed causal framework of the study on maternal intake of vitamin D supplements in mid-pregnancy and pubertal timing in the children; Supplementary Table S1: Maternal intake of vitamin D according to participation in the Puberty Cohort; Supplementary Table S2: Maternal intake of vitamin D supplements in mid-pregnancy according to maternal early pregnancy supplement intake; Supplementary Text S1: Correlation between self-reported intake of vitamin D and plasma 25(OH)D_3._
