# Supplementary material for: Maternal Intake of Vitamin D Supplements during Pregnancy and Pubertal Timing in Children: A Population-Based Follow-Up Study
Source: Nutrients. 2023 Sep 18;15(18):4039. doi: 10.3390/nu15184039 (PMC10536415; doi:10.3390/nu15184039)
Supplement: Supplementary file 1 [file nutrients-15-04039-s001.zip › nutrients-2601122-supplementary.pdf]

Supplementary material

Page 2: Supplementary Figure S1: Directed acyclic graph illustrating the assumed causal framework of the study on maternal intake of vitamin D supplements in mid-pregnancy and pubertal timing in the children.

Page 3: Supplementary Table S1: Maternal intake of vitamin D according to participation in the Puberty Cohort

Page 4: Supplementary Table S2: Maternal intake of vitamin D supplements in mid-pregnancy according to maternal early pregnancy supplement intake

Page 5: Supplementary Text S1: Correlation between self-reported intake of vitamin D and plasma 25(OH)D_3

Supplementary Figure S1: Directed acyclic graph illustrating the assumed causal framework of the study on maternal intake of vitamin D supplements in mid-pregnancy and pubertal timing in the children.

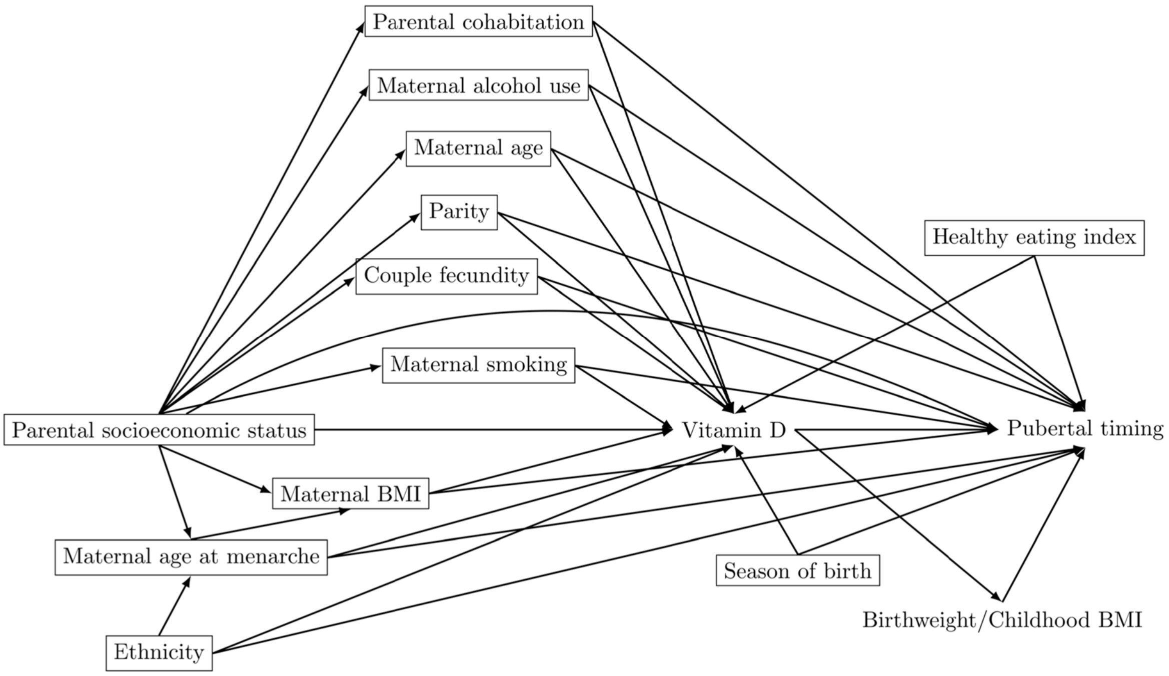

Abbreviations: BMI, body mass index

Boxes indicate conditioning. Ethnicity was adjusted for by design, since the Danish National Birth Cohort consisted of primarily Caucasians. Healthy eating index was adjusted for in a sensitivity analysis. The potential mediation of birthweight and childhood BMI was investigated in sensitivity analyses.

Supplementary Table S1: Maternal intake of vitamin D according to participation in the Puberty Cohort

Maternal intake of vitamin D supplements according to participation in the Puberty Cohort for 17,392 participants with information from the food frequency questionnaire in mid-pregnancy, Denmark, 2000 – 2021

	Participants	Non-participants
	n = 12,991	n = 4,401
Mean vitamin D supplements in µg/day (SD)	7.6 (0.04)	7.9 (0.08)
Mean vitamin D from foods in µg/day (SD)	3.6 (0.02)	3.5 (0.04)
Prevalence of early pregnancy vitamin D supplement intake	10.6%	10.2%

Abbreviations: SD, Standard deviation

Supplementary Table S2: Maternal intake of vitamin D supplements in mid-pregnancy according to maternal early pregnancy supplement intake

Distribution of maternal intake of vitamin D supplements (µg/day) in mid-pregnancy according to maternal early pregnancy supplement intake among 12,991 children from the Puberty Cohort, Denmark, 2000 – 2021

Early pregnancy vitamin intake	Mean maternal intake of vitamin D supplements in µg/day (SD) in mid-pregnancy
Vitamin D with/without calcium	9.1 (6.1)
Multivitamin	7.9 (4.1)
Other vitamin	5.4 (4.6)
No vitamin	6.4 (4.7)

Abbreviations: SD, Standard deviation

Supplementary Text S1: Correlation between self-reported intake of vitamin D and plasma 25(OH)D_3

It was possible to assess the correlation between self-reported intake of vitamin D and 25(OH)D_3 in The Fetal Programming of Semen Quality (FEPOS) cohort, which represents a sub population of the Danish National Birth Cohort (DNBC). The FEPOS is a nested cohort in the DNBC. There were no overlap between FEPOS and the Puberty Cohort.

The FEPOS cohort was established as a male-offspring cohort within the DNBC in 2017 (1). If the mothers had completed the first two questionnaires in the DNBC in addition to having a gestational blood sample stored in the biobank, sons aged 18 years and 9 months and living in the area of Aarhus or Copenhagen were eligible for invitation. Between March 2017 and December 2019, 5,697 adult sons born 1998 – 2000 were invited among the 21,623 eligible for participation in FEPOS. In total, 1,058 sons participated (19%). Further details are described in the cohort profile paper (2).

Maternal 25(OH)D_3

The plasma from the gestational blood samples obtained from the mothers of the sons included in FEPOS was stored at -80°C in the Danish National Biobank, Copenhagen, Denmark. As some cryotubes contained too little plasma for analysis, a total of 827 plasma samples was analysed for 25-hydroxyvitamin D_3 (25(OH)D_3) from 2019 – 2020.

Quantitative analysis of 25(OH)D_3 was performed using two-dimensional liquid chromatography tandem mass spectrometry (LC-MS/MS; QTRAP 6500+; AB Sciex, Framingham, MA, USA) at the Division of Occupational and Environmental Medicine, Lund University. The method is previous described in detail (3). Quality controls met given standards.

Correlation between self-reported intake of vitamin D and 25(OH)D_3

Ordinary least square linear regression models and Pearson's correlation coefficient with 95% confidence intervals (CIs) were used to assess the correlation between maternal 25(OH)D_3 level in plasma and self-reported information on i: intake of vitamin D from supplements; ii: intake of vitamin D from diet; and iii: total dietary intake of vitamin D. For a subset of the mothers having information on gestational 25(OH)D_3 level, we had self-reported information on intake of vitamin D from diet and from dietary supplementations (n = 598). This information

was derived from the food-frequency questionnaire (FFQ) provided in gestational week 25, as described in the main text.

A higher intake of vitamin D from supplements was associated with a higher maternal plasma level of 25(OH)D_3 of 0.79 (95% CI: 0.36; 1.23) nmol/L/µg/day. Pearson's correlation coefficient was 0.14 (95% CI: 0.06; 0.21). A higher intake of vitamin D from diet was not associated with a higher maternal plasma level of 25(OH)D_3 (0.0 (95% CI: -0.61; 0.52)). Pearson's correlation coefficient was 0.0 (95% CI: -0.08; 0.08). A higher total dietary intake of vitamin D from diet and supplement combined was associated with a higher maternal plasma level of 25(OH)D_3 of 0.46 (95% CI: 0.12; 0.80) nmol/L/µg/day. Pearson's correlation coefficient was 0.11 (95% CI: 0.03; 0.18).

Supplements with vitamin D may therefore correlate better with bioavailable vitamin D than vitamin D from diet as reported in the FFQ in this population.

References

1. Olsen J, Melbye M, Olsen SF, Sorensen TI, Aaby P, Andersen AM, et al. The Danish National Birth Cohort--its background, structure and aim. Scand J Public Health. 2001; 29:300-307.

2. Keglberg Hærvig K, Bonde JP, Ramlau-Hansen CH, Toft G, Hougaard KS, Specht IO, et al. Fetal Programming of Semen Quality (FEPOS) Cohort - A DNBC Male-Offspring Cohort. Clin Epidemiol 2020: 757-770.

3. Gaml-Sørensen A, Brix N, Hærvig KK, Lindh C, Tøttenborg SS, Hougaard KS, et al. Maternal vitamin D levels and male reproductive health: a population-based follow-up study. Eur J Epidemiol. 2023.
